# Predicting multiphase flow and tracer transport for an underground chemical explosive test

**DOI:** 10.1038/s41598-026-35868-w

**Published:** 2026-02-17

**Authors:** John P. Ortiz, Dolan D. Lucero, Esteban Rougier, Earl E. Knight, S. Michelle Bourret, Bradley G. Fritz, Miles A. Bodmer, Jason E. Heath, Chelsea W. Neil, Hakim Boukhalfa, Kristopher L. Kuhlman, Shawn Otto, Souheil Ezzedine, Barry L. Roberts, R. Charles Choens, George A. Zyvoloski, Philip H. Stauffer, PE1 Experimental Team, George Abbott, Thomas Alexander, Ethan Alger, Adan Alvarez, Tarabay Antoun, Graham Auld, Hector Banuelos, Marcus Barela, Tyler Barnhart, Perry Barrow, Tara Bartlett, Arturo Bockman, Miles Bodmer, Kyren Bogolub, Jesse Bonner, Rose Borden, Hakim Boukhalfa, Danny Bowman, Carl Britt, Benjamin Broman, Scott Broome, Brian Brown, Jeff Burghardt, Daniel Chester, R. Charles Choens, Kirsten Chojnicki, Al Churby, Justin Cole, Thomas Coleman, Jon Collard, Alexander Couture, Glenn Crosby, Alvaro Cruz-Cabrera, Musa Dea, Walter Dekin, Beirl De Visser, Matthew Dietel, Christine Downs, Nicholas Downs, Damien D’Saint Angelo, Elizabeth Dzenitis, Eric Eckert, Stephanie Eras, Garrett Euler, Souheil Ezzedine, Jose Falliner, Jim Fast, Kristine Featherston, Joshua Feldman, Michael Foxe, Clayton Freimuth, Brad Fritz, Sergio Gamboa, Lisa Garner, Thomas Gascoigne, Jason Gastelum, Jessie Gaylord, David Gessey, Brian Glasgow, Graham Glavin, Andrew Glomski, Matthew Goodwin, David Green, James Griego, Scott Grover, Jose Madrid Gutierrez, Derek Haas, Rodger Hall, Allyson Hall, Daniel Hardy, Dylan Hauk, Jason Heath, James Holdcroft, Austin Holland, Will Honjas, Kaleb Howard, Clayton Hudson, Matthew Ingraham, Johnny Jaramillo, Ayrton Jenkins, Christine Johnson, Kyle Jones, William Junor, Martin Keillor, Graham Kent, Michael Keogh, Will Kibikas, Kieran Kleadbeater, Hunter Knox, James Knox, Kristopher Kuhlman, Christopher Kwiatkowski, Ken Laintz, Joey Lapka, Jennifer Larotonda, John Layne, Pierre-Yves Le Bas, Nick Ledoux, Shengtai Li, Dorothy Linneman, Paul Lipkowitz, Gordon MacLeod, Amrit Malach, Erin McCann, Ryan McCombe, Collin Meierbachtol, Rob Mellors, Brian Memmott, Wade Mendenhall, Jennifer Mendez, Xavier Miller, Andrew Miller, Francisco Miranda, Manny Montano, Michael Moore, Joseph Morris, William Munley, Edgar Godoy Murillo, Steve Myers, Taylor Myers, Annabelle Navarro, Stuart E.J. Nippress, Shawn Otto, Sheila Peacock, Steve Pemberton, Rose Perea, Jacob Peterson, Gabe Plank, Agatha Podrasky, David Podrasky, Joseph Pope, Mike Poskey, Matthew Powell, Amanda Price, Andrew Puyleart, Bobby Quintana, Thom Rahn, Carlos Rendon, Justin Reppart, Hernan Rico, Barry Roberts, Eric Robey, Rebecca Rodd, Mark Rodriguez, Aaron Rogall, Alexander Romanczuk, Melissa Roth, George Salyer, Bill Savran, Walter Schalk, Cari Seifert, Daniel Seitz, Xuan-Min Shao, Dana Sirota, Johnathan Slack, Dave Slater, Ken Smith, Devon Smith, Brady Spears, Dale Sprinkle, Philip Stauffer, Richard Stead, Mary Stephens, Chris Strickland, Alex Tafoya, Joshua Tafoya, M’balia Tagoe, Chad Taguba, Liane Tarnecki, Rees Tatge, Stephanie Teich-McGoldrick, Ben Terry, Ryan Thompson, Margaret Townsend, Greg Tubbs, Reagan Turley, Nichole Valdez, Aaron Van Morris, Sergio Vergara, Juan-Antonio Vigil, Javier Villanueva, Oleg Vorobiev, Darrin J. Wallace, Tim Walrath, Sonia Wharton, Robert White, Helen White, Aliya Whitehill, Marc Williams, Jennifer Wilson, Lynn Wood, Cliff Wright, Andrew Wright, Guangping Xu, Xianjin Yang, Ray Yost, Cleat Zeiler

**Affiliations:** 1 Los Alamos National Laboratory, Los Alamos, NM USA; 2 Pacific Northwest National Laboratory, Richland, WA USA; 3 Sandia National Laboratories, Albuquerque, NM USA; 4 Lawrence Livermore National Laboratory, Livermore, CA USA; 5 Neptune and Company, Los Alamos, NM USA; 6 Atomic Weapons Establishment, Berkshire, England; 7 Mission Support and Test Services, North Las Vegas, NV USA; 8 Nevada National Security Site, Las Vegas, Nevada USA; 9 National Oceanic and Atmospheric Administration, Silver Spring, MD USA; 10 University of Nevada, Reno, NV USA; 11 University of Texas at Austin, Austin, TX USA

**Keywords:** Engineering, Physics

## Abstract

Detecting radionuclide gas seepage from clandestine underground nuclear tests is central to nonproliferation explosion monitoring research. Yet, early-time (<6 day) gas transport driven by the explosive pressure wave remains poorly constrained due to scarcity of field data. We simulate multi-phase gas transport in the vadose zone using pre-shot data from a recent chemical explosion in P-Tunnel at the Nevada National Security Site, USA. Despite using a simplified 2D-radial model, predictions of tracer arrival matched observations within one order-of-magnitude. Our results show how transient blast forcing rapidly mobilizes gases from the cavity into surrounding rock – critical for optimizing sensor placement and test planning. This unique integration of field data and modeling represents a significant improvement in our ability to predict gas migration from underground explosions. More broadly, it offers insights into the coupled dynamics of pressure waves and contaminant transport in the vadose zone, with implications for monitoring and hazard assessment.

**Supplementary Information:**

The online version contains supplementary material available at 10.1038/s41598-026-35868-w.

## Introduction

Detonated underground nuclear explosions (UNEs) produce radionuclide gases such as radioxenon (Xe) that may seep to the surface weeks to months following an explosion. Detection of specific radioisotopes in the atmosphere is considered a “smoking gun” that a nuclear test took place^1,2^. This makes understanding the migration of UNE-related radionuclide gases a vital aspect of nuclear explosion monitoring research and development.

Late-time (weeks to months) seepage of gases to the atmosphere is primarily controlled by a barometrically-driven mechanism referred to as “barometric pumping”^3–6^. Barometric pressure fluctuations pull gases towards the land surface during periods of decreasing pressure and push gas into the subsurface when pressure is increasing, coupling advection with a slower adsorption or diffusion mechanism into the matrix creates a ratcheting capable of significantly faster transport than would occur by diffusive transport alone^6–8^, but much slower than early-time cavity-driven flow. The efficiency with which barometric pumping extracts subsurface trace gases^8,9^ following a UNE ultimately controls the detectability of gas signatures at the surface. Barometric pumping starts where early-time gas migration driven by the residual pressure from the explosive blast leaves off. We therefore require a better understanding of how the explosion-induced pressure conveys the tracer gases from the cavity into the surrounding subsurface environment.

Efforts to validate models of early-time mass transport for underground nuclear and chemical explosions have been hindered by a lack of field-scale experimental data that include the relevant processes, such as the explosive pressure wave. Conducting such experiments is complex and is prohibitively expensive for most institutions to perform. Until recently, the community relied on legacy UNE test data (i.e., from before the current nuclear testing moratorium) to inform models. However, although legacy data contains a wealth of information pertaining to many aspects of the nuclear explosion, detailed gas transport observations were not collected. The ongoing Physics Experiment 1 (PE1)^10^ is a series of underground chemical explosion experiments conducted at the United States’ Nevada National Security Site (NNSS) that is designed to fill this and other knowledge gaps. Quantification of early-time transport processes, key to gas migration monitoring efforts, can be achieved by validating sophisticated hydrogeologic flow and transport models against data from physically relevant field experiments such as PE1.

In this study, we present the results of predictive flow and transport simulations we performed during the planning stages of the PE1 experiment using hydrogeologic data calibrated before the shot^11^. The gas transport predictions are then compared to the experimental results. For the predictions, we simulate multi-phase (gas- and aqueous-phase) vadose zone transport of a radionuclide tracer (xenon) and high-explosive (HE) byproducts resulting from early-time pressure-wave propagation following an underground chemical explosion. Cavity fluid pressure (>14 MPa) and temperature (>1300 °C) conditions created by the explosion are much higher than conditions usually encountered in subsurface flow and transport models above the water table, so we required modifications to our flow and transport simulator to handle the conditions^12^. We incorporate predictions of inferred permeabilities and pressure propagation^11^ into a numerical flow and transport model and compare simulated tracer concentration predictions to measurements from borehole gas samplers surrounding the explosion cavity. Uncertainty quantification of hydraulic properties is included in our prior estimates of several relevant gas transport properties. Although our model necessarily relies on simplifying assumptions and idealized boundary conditions, it reproduces the measured borehole concentrations with reasonable, order-of-magnitude accuracy at several locations using the pre-shot permeability estimates*.* Less accurate fits are observed at more distal boreholes, reflecting the sensitivity of the predictions to spatial heterogeneity and the limitations of pre-shot calibration data. This research integrates a unique dataset with numerical modeling to improve our understanding of the explicit connection between pre-shot hydrogeologic characterization and the resulting early-time transport of gas tracers in response to an underground chemical explosion.

## Methods

### Experiment description

**Fig. 1:**
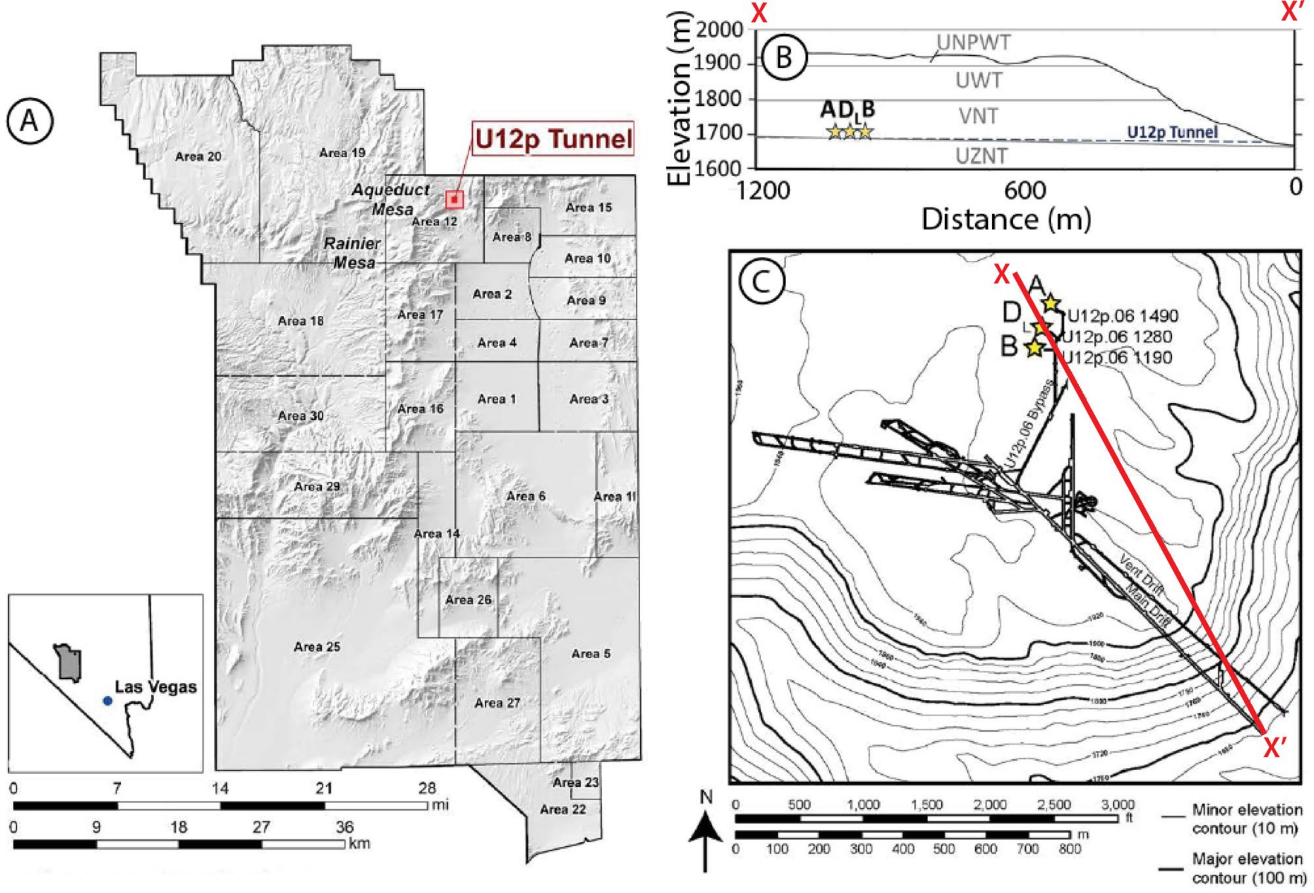
Map and cross-section views of the testbed within the study area. (**a**) Location of the Nevada National Security Site, Aqueduct Mesa (red box) which houses U12p Tunnel (P-Tunnel). (**b**) Cross section of Aqueduct Mesa from X – X’ in (**c**). The layers correspond to the following lithologies: upper nonwelded to partially welded tuff (UNPWT), upper welded tuff (UWT), vitric nonwelded tuff (VNT), and upper zeolitic nonwelded tuff (UZNT). (c) Topographic contours of Aqueduct Mesa and P-Tunnel Complex. Yellow stars represent the locations of the Physics Experiment 1 (PE1) chemical explosion experiments (PE1-A, -B, and -D_L_). Figure modified from^10^, U.S. Department of Energy technical report prepared by Lawrence Livermore National Laboratory under Contract DE-AC52-07NA27344. Original map created using digital elevation model data within the Generic Mapping Tools (GMT) software^14^.

To better understand the migration of gases driven by the explosive pressure wave, a series of underground chemical explosions are being conducted at the NNSS as part of PE1^10^. These experiments are designed to allow real-time monitoring of pressure propagation and gas migration in the rocks surrounding the cavity following the explosion. The P-Tunnel complex, which hosts the three testbeds in primarily volcanic tuff geology, is located within Aqueduct Mesa in the NNSS^13^ (Fig. 1). PE1-A was the first experiment in the series and was performed in October 2023. Instrumentation for PE1-A included eight gas sampling (GS) boreholes at varying distances from the cavity working point at elevations both above and below the working point elevation. Pressure transducers were co-located. Data from these transducers were previously used to characterize the hydraulic properties (e.g., permeability) of the surrounding rock using pre-shot cavity pressurization data^11^.

### Site description and hydrogeologic setting

**Fig. 2:**
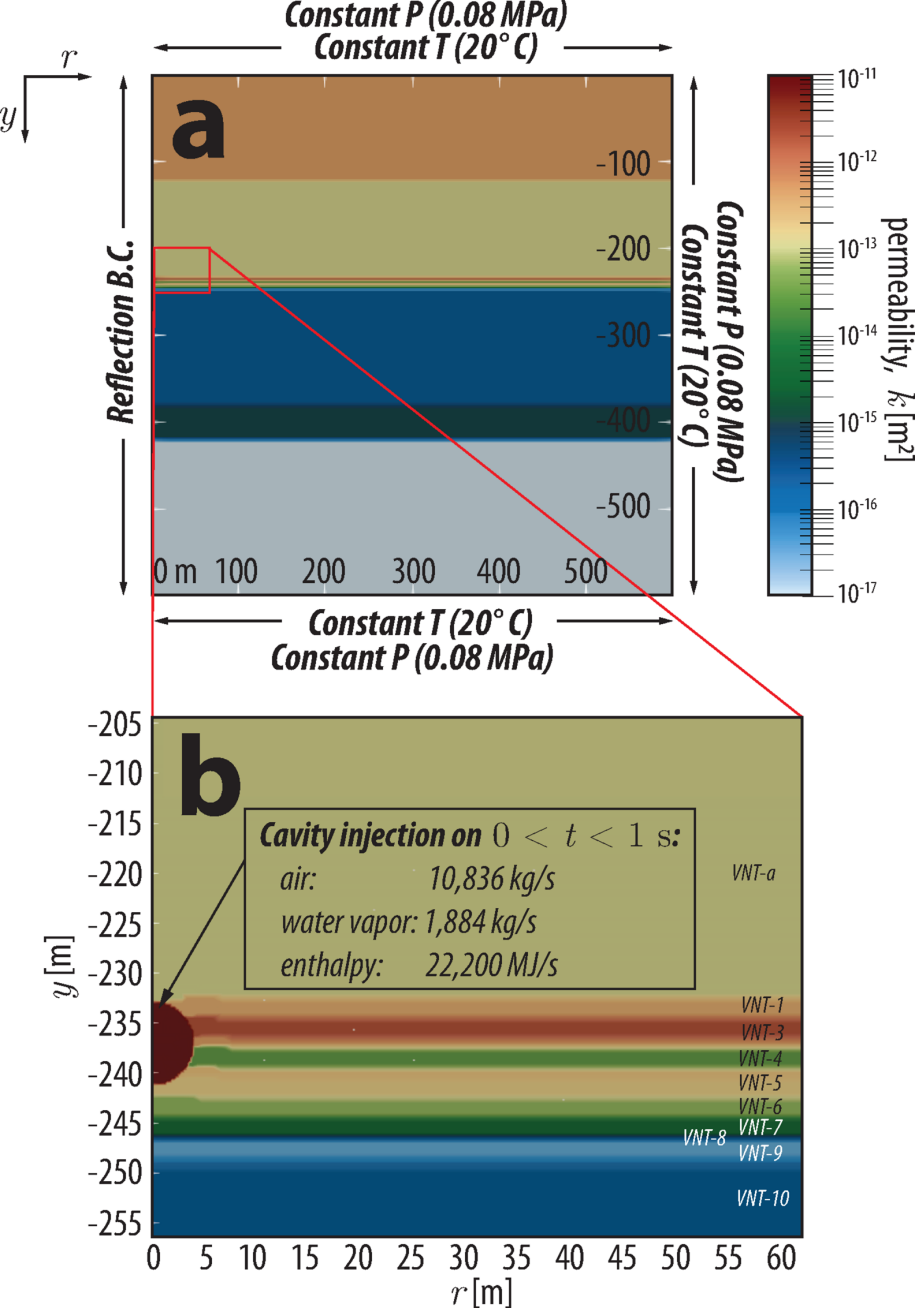
Schematic of the hydrogeologic framework of the model and boundary conditions for (**a**) the full extent of the model domain, and (**b**) inset zoomed into region surrounding the cavity. The injection source terms are distributed uniformly throughout the cavity volume. VNT units in (b) correspond to those in Table 1.

**Fig. 3:**
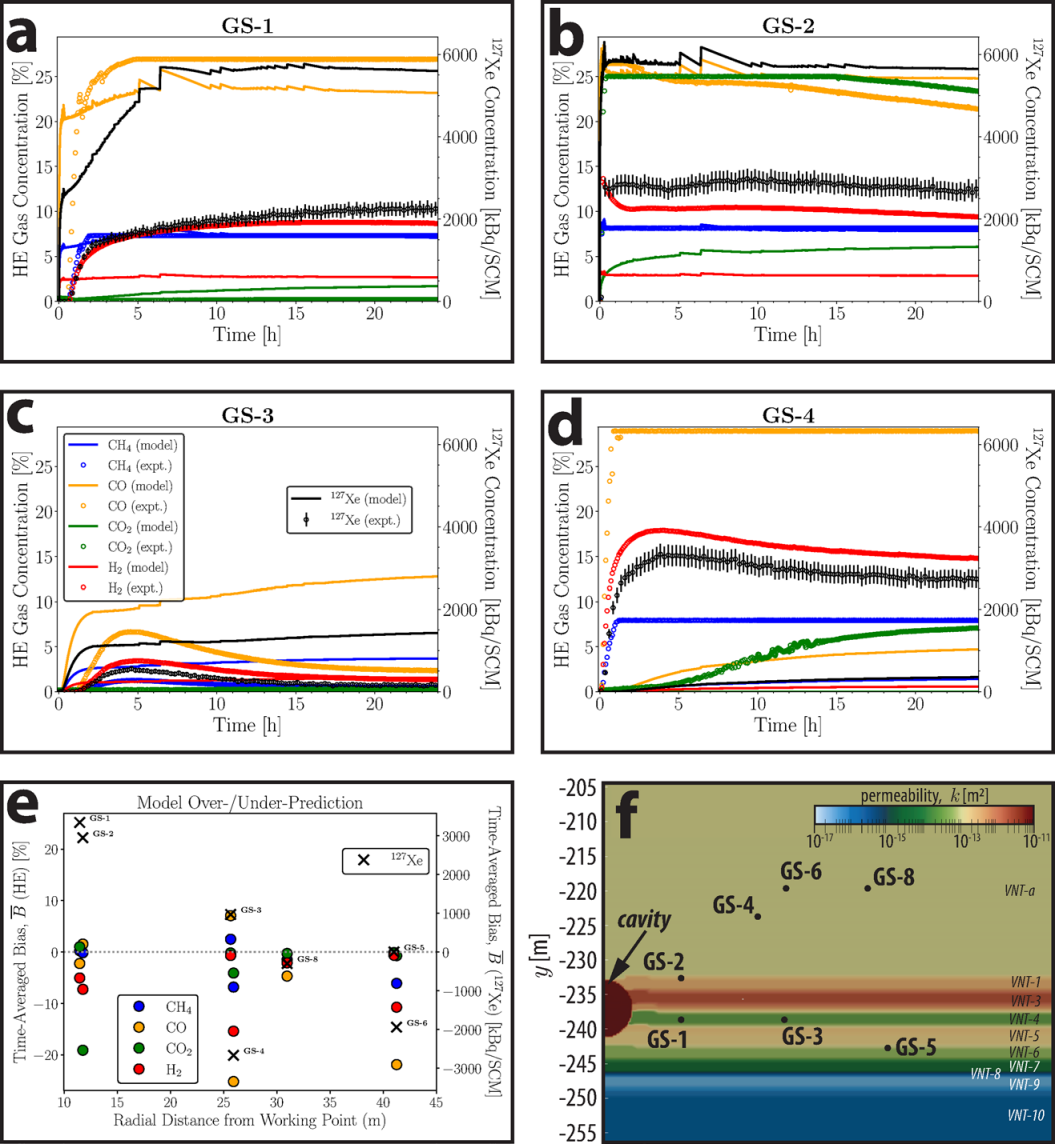
Composite plot of simulated gas concentrations using permeability values from pre-shot and experimental gas concentrations at proximal boreholes: (**a**) GS-1, (**b**) GS-2, (**c**) GS-3, (**d**) GS-4; (**e**) bias (over-/under-prediction; Equation 9) plotted as a function of total distance from the working point and (**f**) zoomed-in section of model domain showing GS borehole locations. Note that for (a-e) HE byproducts concentrations/bias are represented by circles corresponding to the left *y*-axis [%] scale, whereas ^127^Xe is represented by black “x” markers corresponding to the right *y*-axis [kBq/SCM] scale. Boreholes GS-6 and 8 are not shown because simulated concentrations are orders of magnitude lower than observed. Borehole GS-5 is not shown because breakthrough concentrations reported by both the experiment and model were negligible.

The geology hosting the P-Tunnel complex is primarily composed of pumice- and ash-fall and ash flow deposits (Timber Mountain Formation; TM)^15^. Some tuffs have been reworked and exhibit various degrees of welding and zeolitization, however, much of the volcanic lithology has experienced little structural deformation from Basin and Range Extension^16^. P-Tunnel is approximately 350 m above the water table^17^. Figure 2a shows the lithology of the Aqueduct Mesa, where P-Tunnel resides. From the surface to 400 m depth, the lithology is as follows: upper nonwelded to partially welded tuff (UNPWT), upper welded tuff (UWT), vitric nonwelded tuff (VNT) and upper zeolitic nonwelded tuff (UZNT). The PE1-A cavity is positioned within the transition zone where nonwelded tuff becomes progressively more zeolitized with increasing depth as it grades downward into the UZNT (Fig. 2b, Fig. 3f). Eleven geologically distinct subunits have been identified within the VNT units exposed by P-Tunnel excavation. The units are ordered from shallowest to deepest, with VNT-a lying above numbered VNT units.

A series of boreholes (GS-1 to 8) were drilled in the drifts to characterize the inter- and intra-subunit heterogeneity in the VNT^18^. Core and grab sample analysis is described in detail in previous studies^19,20^, which characterized hydraulic and physical properties such as saturation, density, porosity, and relative permeability. Because of the scale-dependence of permeability, further calibration of the permeability was required. Core and grab sample data are used to inform the calibration using pressure data collected during a pre-shot cavity pressurization test^11^. These pre-shot calibrated permeabilities are used in the present study and are presented along with the other hydraulic properties used in Table 1.

**Table 1:** Hydraulic properties for simulated units/features. Pre-shot permeability values are calibrated via cavity pressurization test^11^. Post-shot permeabilities are calculated as an exercise using pressures from observations of PE1-A following the methodology described in previous work^11^. Units are fully described in^19,20^. The average saturation (0.65) from other VNT units is used for VNT-1, 6–10.

**Geologic Unit/** **Feature**	**Co-located** **Borehole ID**	**(Pre-shot)** **Permeability** **[m**^**2**^**]**	**(Post-shot)** **Permeability** **[m**^**2**^**]**	**Porosity** **[–]**	**Water** **Saturation** **[–]**	**Description**
VNT-a	GS-4,6,8	9.86 $\cdot$ 10^−14^	5.00 $\cdot$ 10^−13^	0.36	0.65	Vitric
VNT-1	GS-2	3.84 $\cdot$ 10^−13^	4.62 $\cdot$ 10^−12^	0.36	0.65	Vitric
VNT-3	GS-7	2.13 $\cdot$ 10^−12^	2.34 $\cdot$ 10^−13^	0.35	0.43	Vitric, becoming zeolitic and silicified lower
VNT-4	–	4.99 $\cdot$ 10^−13^	1.03 $\cdot$ 10^−14^	0.30	0.65	Mostly zeolitic, vitric at top and partially vitric at base
VNT-5	GS-1,3	2.59 $\cdot$ 10^−14^	4.81 $\cdot$ 10^−15^	0.37	0.76	Mostly vitric, with some partially zeolitic intervals
VNT-6	–	2.42 $\cdot$ 10^−13^	3.00 $\cdot$ 10^−14^	0.32	0.65	Mostly vitric, grading to more zeolitic lower
VNT-7	–	1.74 $\cdot$ 10^−13^	2.68 $\cdot$ 10^−14^	0.24	0.65	Mostly zeolitic, locally vitric
VNT-8	GS-5	5.84 $\cdot$ 10^−14^	4.43 $\cdot$ 10^−16^	0.32	0.65	Mostly vitric, with zeolitic alteration grading in and out
VNT-9	–	2.79 $\cdot$ 10^−15^	1.09 $\cdot$ 10^−15^	0.24	0.65	Mostly vitric, with zeolitic alteration grading in and out.
VNT-10	–	3.43 $\cdot$ 10^−17^	7.52 $\cdot$ 10^−17^	0.16	0.65	Mostly vitric with weak zeolitic and local alteration
Cavity	–	1.00 $\cdot$ 10^−11^	2.64 $\cdot$ 10^−10^	0.999	0.00	–

### Numerical multiphase subsurface flow and transport simulations

Numerical calculations are performed using the Finite-Element Heat and Mass (FEHM) simulator, a well-tested multiphase code^21–23^. FEHM has been used extensively in subsurface flow and reactive transport studies of radionuclide gases^5,8,24–29^. Hydraulic properties (e.g., permeability, porosity) of rock units within our hydrogeologic framework were calibrated using pre-shot cavity pressure test data in recent work^11^. We simulate gas transport, driven by explosion-induced overpressures in the cavity, and compare time-varying concentrations at borehole locations to measured gas sampler concentrations. The model is set up in a two-dimensional axially symmetric (cylindrical) geometry that assumes radial symmetry about the *y*-axis because it is dimensionally consistent with the experiment in terms of volume and overall depth of the study area. The computational mesh was generated using the Los Alamos Grid Toolbox (LaGriT)^30^.

#### Governing equations for flow and tracer transport

The governing flow equations solved by FEHM in this study for multi-phase flow in unsaturated porous media are given by:

1 $$\frac{\partial }{\partial t}\left[{{S}_{i}\phi \rho }_{i}\right]+\nabla \cdot \left({\rho }_{i}{\overrightarrow{q}}_{i}\right)={Q}_{i}\left(t\right),\text{ where}$$

2 $${\overrightarrow{q}}_{i} =-\frac{k{k}_{r}\left({S}_{i}\right)}{{\mu }_{i}}\nabla {P}_{i}$$

where ${S}_{i}$ is saturation [dimensionless] of a given fluid phase *i* (either gas *g* or liquid *l*), given by ${S}_{i}={V}_{i}/{V}_{void}$ where $V$ is volume [m^3^]; $\phi$ is porosity [dimensionless]; ${\rho }_{i}$ is the given fluid density [kg/m^3^]*;* ${\overrightarrow{q}}_{i}$ is volume flux per unit area [m^3^/(m^2^$\cdot$s)] of the given phase; ${\mu }_{i}$ is viscosity [Pa $\cdot$ s]; $t$ is time [s]; $k$ is intrinsic rock permeability [m^2^]; ${k}_{r}\left({S}_{i}\right)$ is the relative permeability [dimensionless] of each phase; ${P}_{i}$ is pressure [Pa]; and ${Q}_{i}\left(t\right)$ is the source term [kg/(m^3^$\cdot$s)] for a given phase. Here, we use a multimodal van Genuchten relative permeability model^31^ implemented in a previous study^11^. The non-linearity is a result of the pore distribution, a combination of matrix pores and pre-existing or post-explosion microfractures in VNT units. We assume that the bulk movement of fluids through the rock matrix behaves according to Darcy’s law (Equation 2).

The governing transport equations solved by FEHM for conservative gas tracers are given by the conservative advection-diffusion equations:

3 $$\frac{\partial }{\partial t}\left[\left({S}_{g}\right)\phi {\rho }_{g}C\right]= -\nabla \cdot \left({\rho }_{g}\overrightarrow{q}C\right)+\nabla \cdot \left(\phi {\rho }_{g}{D}_{g}\nabla C\right)$$

where ${S}_{g}$ ($=\left(1-{S}_{l}\right)$) is the saturation of the gas phase [dimensionless], $C$ is the tracer concentration [mol/kg_air_], ${D}_{g}$ is the molecular diffusion coefficient of the given gas species in air [m^2^/s], the subscript *g* indicates the gas phase, and the other variables are as previously defined.

Gas-phase tracer mass can also partition into the aqueous phase according to Henry’s law assuming equilibrium conditions:

4 $${C}_{g}= \frac{{C}_{aq}}{{k}_{H}^{\circ }}\frac{1}{RT{\rho }_{v}} ,$$

where ${k}_{H}^{\circ }$ is the Henry solubility constant in water [mol/(kg⋅bar)], ${C}_{aq}$ is the concentration of the species in the aqueous phase [mol/kg _*w*_], $R$ is the universal gas constant, $T$ is temperature [K], and ${\rho }_{g}$ is gas density [kg/m^3^]. Henry’s law solubility coefficients are given in Table 2. The transport of a given tracer that has partitioned into the aqueous phase is given by:

5 $$\frac{\partial }{\partial t}\left[{S}_{l}\phi {C}_{aq}\right]=\nabla \cdot \left({S}_{l}\phi {D}_{l}\nabla {C}_{aq}\right)$$

where ${S}_{l}$ is the saturation of the liquid phase [dimensionless] and ${D}_{l}$ is the liquid-phase diffusion coefficient [m^2^/s] of the species in excess water. Tracers may dissolve into the aqueous phase and be transported by advection-diffusion, however the borehole sensors only detect gas-phase constituents

**Table 2:** Mass distribution of HE and radionuclide compounds in PE1-A cavity.

**Compound**	${{\boldsymbol{N}}}_{{\boldsymbol{m}}{\boldsymbol{o}}{\boldsymbol{l}}}$	${{\boldsymbol{C}}}_{0}$**[mol/kg**_**air**_**]**	${{\boldsymbol{k}}}_{{\boldsymbol{H}}}^{\circ }$**[mol/(kg**·**bar)]**^32^
CO	1.11 $\cdot$ 10^5^	433.55	9.9 $\cdot$ 10^−4^
CO_2_	8.38 $\cdot$ 10^4^	328.83	3.5 $\cdot$ 10^−2^
CH_4_	4.28 $\cdot$ 10^4^	167.79	1.4 $\cdot$ 10^−3^
H_2_	1.00 $\cdot$ 10^4^	39.40	7.8 $\cdot$ 10^−4^
^127^Xe	3.18 $\cdot$ 10^−7^	1.25 $\cdot$ 10^−9^	4.3 $\cdot$ 10^−3^

The governing equations for conservation of energy in FEHM is given in terms of energy per unit volume (${A}_{e}$) by:

6 $$\frac{\partial {A}_{e}}{\partial t}+\nabla \cdot \overrightarrow{{f}_{e}}+\dot{F}\text{, where}$$

7 $${A}_{e}=\left(1-\phi \right){\rho }_{r}{\gamma }_{r}+\phi ({{S}_{g}{\rho }_{g}{\gamma }_{g}+S}_{l}{\rho }_{l}{\gamma }_{l})$$

with ${\gamma }_{i}={C}_{p,i}T$, and the energy flux $\overrightarrow{{f}_{e}}$ given by:

8 $$\overrightarrow{{f}_{e}} = {\rho }_{g}{h}_{g}{\overrightarrow{q}}_{g}+ {\rho }_{l}{h}_{l}{\overrightarrow{q}}_{l}-\kappa \nabla T$$

where $\overrightarrow{{f}_{e}}$ is energy flux with units of energy per area time [J m^2^/s]; $\dot{F}$ is the energy source term which can be a function of time [J/(m^3^ $\cdot$ s)]; the subscript *r* refers to the solid rock matrix; $\gamma$ is the specific internal energy [J/kg] for each material; ${C}_{p,i}$ is specific heat [J/(kg $\cdot$ K)]; ${h}_{g}$ and ${h}_{l}$ are specific enthalpies [J/kg] for gas and liquid phases, respectively; and $\kappa$ is effective thermal conductivity [W/(m $\cdot$ K)].

#### Boundary and initial conditions

The simulations are run under non-isothermal conditions. Air pressure in the model domain is initially uniform and set to 0.08 MPa, the approximate average background atmospheric pressure at the NNSS. The effects of an air-static gradient in the subsurface and the influence of barometric fluctuations on the ground surface are not considered, as the forcing from the overpressured cavity ($\Delta P$ >14 MPa) overwhelms any local gradients on the time scale of our simulations. Tracer concentrations are initially 0 mol/kg_air_ everywhere except in the cavity. Tracer gases have provenance either as explicit inclusions in the experiment for modeling UNE signatures (xenon-127, ^127^Xe), or as HE byproducts (carbon monoxide, CO; carbon dioxide, CO_2_; methane, CH_4_; and hydrogen, H_2_). Initial gas tracer concentrations in the cavity are known directly for the UNE signatures. Initial concentrations of HE byproducts are calculated from known compositions of the HE source. Initial cavity gas inventory is presented in Table 2.

Constant pressure boundary conditions (Dirichlet) are prescribed on the top, bottom, and right-lateral edge (radial extent) of the model domain, with pressure set to 0.08 MPa (Fig. 2a). The actual ground surface is essentially a time-dependent air pressure boundary; however, due to the short timescales modeled and relatively large depth of the explosion, use of an average air pressure value is sufficient. The constant pressure boundary condition at the lateral boundary represents a far-field boundary and prevents artificial buildup of pressures by allowing water and air to escape. We observed no change in pressure or concentrations when testing on a larger mesh (1000 m $\times$ 1000 m) using identical forcing and boundary conditions. We therefore concluded that the simulation is not affected by boundary effects on the timescale of our model. Tracer mass is allowed to escape on these boundaries by both diffusion and advection along concentration and pressure gradients leaving the model domain. The left lateral edge (*r* = 0 m) is a no-flux/reflection boundary (Neumann).

Cavity pressurization due to the explosion is represented by injecting a combination of non-condensable air and water vapor uniformly distributed within the entire cavity volume for a short interval (1 s) as a specified flow rate (Neumann) boundary condition (Fig. 2b). During this injection interval, air was injected at a rate of 10836 kg/s and water vapor was injected at a rate of 1884 kg/s. Mass of air and water vapor added to the domain are calculated based on the integrated mass of HE byproducts produced by the HE compound used (13850 kg). The simulations do not differentiate different components of the non-condensable gas and simply represent the non-condensable fraction as having the properties of air. We calculate that the density and viscosity of the mixture of combustion by-products are within 10% of air at standard pressure and temperature and recognize that this simplification represents a deviation from the actual system. However, simulating multicomponent non-condensable gas is currently beyond the capability of our numerical code and is a goal for further development. Representing the explosion this way approximates the rapid overpressurization observed and produces peak cavity pressures consistent with those measured during the experiment.

Our gas transport simulations use information from hydrodynamics calculations using the Hybrid Optimization Software Suite (HOSS) multiphysics code^33^ to approximate an early time (1 s) state that maintains consistency between the two models. The hydrodynamic simulations convert high explosives to both pressure and thermal energy and some fraction of the energy is used to compress and damage the rock as the explosion causes the cavity to grow^34^. Thus, in the transport simulations post-detonation, the cavity radius (${r}_{cav}$) is increased from a pre-shot radius of 2 m to approximately 4.4 m. A further modification to the initial state of the transport simulations is the inclusion of a ring of fully saturated (${S}_{l}$ = 1.0) rock of approximately 2 m thickness surrounding the cavity. This saturated ring (Figure S6) represents pore crush seen in the hydrodynamics simulations^35^, which makes the air-filled porosity negligible. Air must then exceed the air entry pressure to force water out of the way and permit gas to escape the cavity.

Thermal loading in the injected air and water vapor is included to match cavity gas inventory estimates of the PE1-A explosion. Those calculations yielded cavity temperatures on the order of 1300 °C at early times. Energy is added to the injected air as enthalpy at a rate of 22200 MJ/s to capture this increase in temperature.

## Results

In general, the simulations using pre-shot diagnostics predict within an order-of-magnitude the gas concentrations at boreholes within about 35 m of the working point; a summary comparison between model and experiment time series is given in Fig. 3. Concentrations at distal boreholes (e.g., GS-6 and 8) are generally underpredicted and are not shown in Fig. 3 – a full visual summary of tracer results for each borehole can be found in the Supporting Information (SI) (Figure S2). Predicted breakthroughs tend to occur sooner than what is observed in the data, but the concentrations generally level out at similar values. One exception is the ^127^Xe tracer, for which the simulated concentrations at proximal boreholes (GS-1 to 3) are much higher than the observed. Simulated gas concentrations for all species are underpredicted at shallow distal boreholes (GS-4, 6, and 8; Fig. 3d, Figure S1f-g). Transport of CO_2_ is also not reliably predicted by the model; concentrations are overpredicted at GS-1 and greatly underpredicted at GS-2. We discuss this in section *4.4 Influence of Adsorption on Tracer Transport*. To visualize prediction performance, we present time-averaged model bias as a function of total distance from the working point in Fig. 3e to provide a sense of where the simulations over- or under-predict gas transport for each species. We calculate this time-integrated bias as below:

9 $$\overline{B }= \frac{{\sum }_{i=1}^{N}\left({y}_{model, i}-{y}_{exp,i}\right)\cdot\Delta {t}_{i}}{{\sum }_{i=1}^{N}\Delta {t}_{i}},$$

where $\overline{B }$ is the time-averaged model bias, ${y}_{model, i}$ is the model value at time step $i$, ${y}_{exp, i}$ the experimental (observed) value at time step $i$, $\Delta {t}_{i}$ is the time interval (duration) associated with step $i$, and $N$ is the number of time intervals. As calculated, Equation 9 is effectively a weighted mean, where the weights are the time durations $\Delta {t}_{i}.$ The bias has the same units as the reported measurements and has negative values for under-prediction, positive values for over-prediction.

## Discussion

The ability of the model to accurately predict gas tracer transport varies significantly across the sensor network and between tracers, with spatial and stratigraphic relationships playing a critical role in prediction accuracy. Here we discuss some of the factors affecting prediction accuracy.

### Sensitivity of model permeability calibration to distance from the working point

There is a clear change in model bias towards underprediction in going from GS-3 to GS-4, despite being very similar in terms of total distance from the working point (Fig. 3e). This transition highlights a trend observed for all the gases, which is that the model underpredicted concentrations for all gas species at boreholes located in VNT-a (i.e., GS-4, 6, and 8). VNT-a is above the working point elevation and is essentially beyond the influence of the cavity pressurization test used to calibrate permeabilities^11^. One conclusion of that study was that the small overpressure ($\Delta P\sim$ 0.07 MPa) created by the cavity pressurization test was unable to perturb the pressure field at shallower distal boreholes (GS-4, 6, and 8), possibly because VNT-a has a much higher permeability than what could be calibrated using pre-shot data. However, the pressure signals at proximal boreholes (e.g., GS-1 and 2) were very sensitive to VNT-a permeability, which constricted the calibration of VNT-a in the absence of perturbed pressure sensors. This provides a reasonable explanation for why the model underpredicts concentrations for all tracer gases at boreholes in this unit. Furthermore, as will be discussed later in this section, VNT-a is almost entirely vitric and non-zeolitic, so tracer-specific preferential adsorption is not likely to be a factor contributing to misfit for these shallower boreholes.

**Fig. 4:**
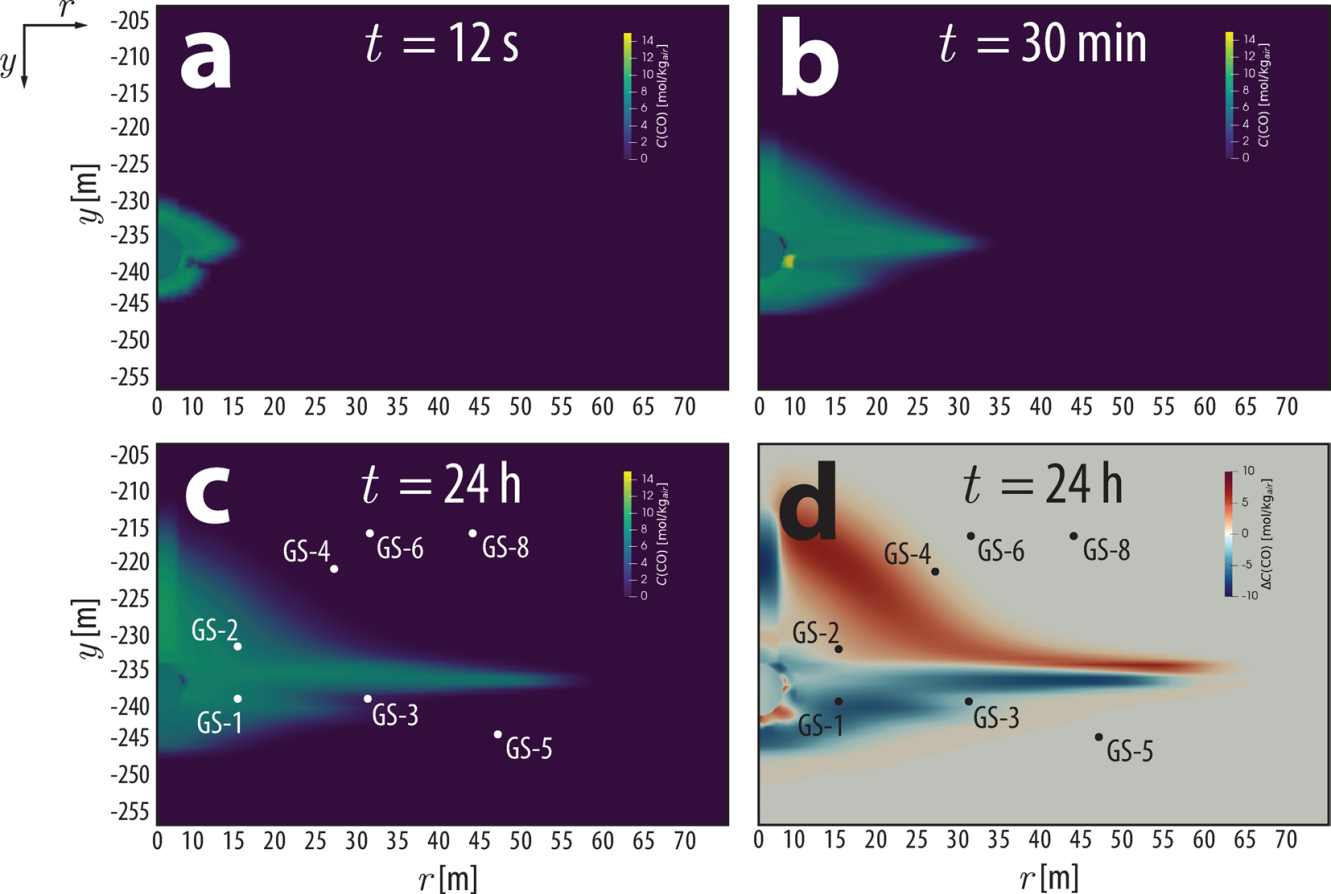
Time slices of modeled CO gas transport plumes at: (**a**) 12 s, (**b**) 30 min, (**c**) 24 h, and (**d**) a comparison ($\Delta {C}_{model}$) of the CO gas plumes by calculating the difference between simulated results using the pre-shot and post-shot calibrated permeabilities, $\Delta {C}_{model}={C}_{post}\left(t\right)-{C}_{pre}\left(t\right)$.

To confirm this hypothesis, we re-calibrated the VNT unit permeabilities using the pressure response data from the PE1-A explosion (i.e., post-shot data) using the same approach described previously^11^. The experiment produced much greater excess cavity pressures ($\Delta P>$ 14 MPa) than what was produced by the pre-shot cavity pressurization, so this approach has the clear benefit of generating pressure perturbations at distal boreholes (e.g., GS-4 to 8) that were unperturbed in the pre-shot pressurization, though such data were clearly not available for the prediction simulations. Key differences resulting from using PE1-A data for calibration include higher calibrated permeabilities in VNT-a, −1, −3, and the cavity, and lower permeabilities in VNT-4 to 10. The pre- and post-shot calibrated permeabilities are both included in Table 1. As might be expected, simulated transport using post-shot calibrated permeabilities more accurately represent the transport observed in the experiment for most species, especially for more distal boreholes (Figure S3). In particular, ^127^Xe concentrations at every borehole location are much closer to those observed (Figure S4). A time slice contour at *t =* 24 h depicting $\Delta {C}_{model}$, which we define as the difference between the modeled gas concentrations produced using the pre- and post-shot calibrated permeabilities ($\Delta {C}_{model}={C}_{post}\left(t\right)-{C}_{pre}\left(t\right)$) is shown in Fig. 4d for CO, which highlights regions where the transport differed depending on the permeabilities used. As was previously hypothesized^11^, the permeability of VNT-a is higher than the values calibrated using the cavity pressurization data because the pre-shot test did not perturb pressures at GS-4 to 8. Higher permeability in VNT-a facilitates increased transport to co-located boreholes (GS-4 to 8) during PE1-A. As a corollary effect, this may have also helped to bleed off excess pressures that had previously been delivered to down-section units (VNT-1 to 10), resulting in lower ^127^ Xe concentrations that more closely match the observations.

The model clearly benefits from using permeabilities we calibrate from data where larger pressure perturbations are generated at a more relevant scale (i.e., the post-shot data), as is reflected in the reduced total error (Figure S4). We did not have such data at the time of our predictive modeling, but it is nonetheless important to note the impact of using parameters calibrated from data collected under different conditions. For VNT-a, the post-shot calibrated permeability returned a higher permeability value (5.0 $\cdot$ 10^−13^ m^2^) than the pre-shot cavity pressurization test calibration (9.9 $\cdot$ 10^−14^ m^2^). The post-shot calibrated permeability for VNT-a more closely resembles the median value obtained from single-hole packer testing (7.2 $\cdot$ 10^−13^ m^2^; minimum and maximum values were 3.3 $\cdot$ 10^−13^ m^2^ and 2.3 $\cdot$ 10^−12^ m^2^, respectively). Both the pre-shot and post-shot calibrated data are essentially cross-hole tests and thus are larger scale measurements than the single-hole packer test data. This result runs somewhat counter to the trend observed previously^11^, wherein generally higher permeabilities were seen as the scale of the observation increased. Because of the scale of the system of interest (i.e., field-scale flow and transport), we would not expect our result to be the case except perhaps at distal boreholes GS-4 through 8 that are not effectively calibrated using data from pressurization tests where the pressure perturbation is too small. This is an important and useful result, as future experiments may not be able to pressurize the cavity during testing to the extent that was possible for PE1-A, such as would be the case for larger cavities and for experiments with more distally located boreholes. Calibration of permeabilities at unperturbed sensors using large-scale pressurization testing may lead to a local minimum, and should instead rely more heavily on smaller-scale measurements (e.g., packer test data).

### Transport heterogeneity and scale effects

A tracer particle traveling to a distal borehole encounters a broader range of geologic heterogeneity compared to one traveling to a proximal borehole. Prior studies have shown that permeability and porosity can be strongly scale-dependent for volcanic tuffs^11,36–40^. These effects are further compounded for resultant transport^41–47^. At field scales (10s–100s of m), transport samples a large range of heterogeneity, leading to greater variability in breakthrough times and concentration distributions at more distant monitoring points. These effects challenge the predictive capability of models like ours that do not explicitly account for spatially heterogeneous transport pathways within layers.

Our model is also limited in how it represents spatial heterogeneity because we have invoked 2-D axi-symmetric domain geometry due to concerns with computational efficiency. Because of this, some gas sampling ports shown in Fig. 3f that appear close to one another (e.g., GS-4 to 8) are in fact radially separated by tens of meters (see Fig. 4 in^10^). This is caused by the translation of actual 3-D space to model 2-D axi-symmetric space and means that the effect of hydrogeologic heterogeneity between such boreholes cannot be directly accounted for in our simulations. The transport simulations inherit the uncertainty associated with this assumption from the pre-shot permeability calibration^11^, which used the same simplifying geometric representation. While it is not possible to fully disentangle the effect of this assumption on the predictions relative to other factors (e.g., insensitivity of distal boreholes to pre-shot permeability calibration), the effect is observed when the model predicts transport with mixed success at two boreholes that appear close in 2-D axi-symmetric space (e.g., GS-4 and 6; Figure S3).

### Effects of water saturation characterization

It is also worth noting limitations regarding characterization of the water saturation variability within the domain. As with permeability, saturation is prescribed uniformly in space within each stratigraphic unit, so the model contains no intra-unit heterogeneity (i.e., heterogeneity within a given unit). Values of saturation are assigned using data collected from core “grab” samples^20^, although VNT-1 and 6 through 10 were not analyzed experimentally and so use an average saturation (0.65) based on the other VNT units. Saturation impacts several factors relevant to tracer transport including solute retardation due to dissolution of vapor-phase tracers, gas and liquid relative permeability, and retardation due to adsorption. We note that the degree of saturation also affects tracer retardation due to adsorption, which was not included in our model, but is discussed in the following section (*4.4 Influence of Adsorption on Tracer Transport*). Retardation of vapor species via Henry’s law dissolution into pore water is described in the Methods section (Equation 4). For a given a set of Henry’s law parameters, higher saturation means a greater quantity of gas tracer can dissolve into pore water, thereby slowing gas-phase transport. Gas and liquid relative permeability affect the ease with which the cavity overpressure is able to move water out of pores in the surrounding rock to permit gas flow. Rock with a higher degree of liquid saturation will have higher liquid relative permeability and lower gas permeability, with the converse having the opposite effect. Because gas permeability is primary control on gas-phase tracer transport, we attempt to justify the estimated saturation values used in our model using previously-performed uncertainty analyses^11^. Those analyses were aimed at determining the impact of saturation ranges found in core analyses with regard to calibrated permeability values, and recalibrated permeabilities within a factor of 2 when using average saturation values. The saturation estimates we use in our model are therefore reasonable given the limited spatial resolution of our saturation measurements.

There is an added layer of complexity in that the saturation in our model is static initially, but is permitted to move within the pore space driven by pressure perturbations. The blast pressure initially pushes the water out of the pores in the fully-liquid-saturated pore crush shell surrounding the cavity, dropping by as much as 40% in some locations and allowing gases to escape. The region just outside the pore crush shell (Figure S6) increases in saturation as water moves in, but the increase in rock volume with radial distance from the originally saturated shell means the increased saturation is unable to fully prevent outward gas movement. There is also some effect of the high-temperature water vapor moving into the surrounding rock, where it cools quickly and condenses, locally increasing saturation. Water readily moves into the highly permeable VNT-3 unit, increasing saturation by 10% as it moves laterally. The units directly neighboring VNT-3 (VNT-1, overlying; VNT-4, underlying) both decrease in saturation by similar amounts. To illuminate these processes, we present time slices of the change in water saturation in the Supporting Information (Figure S5).

### Influence of adsorption on tracer transport

The predictive simulations do not include the effects of adsorption, but recent work^29,48–52^ has demonstrated that adsorption can play a critical role in gas transport within zeolitic tuff. Specifically, CO_2_ adsorption to zeolites in these formations has been shown to be so significant that, under certain conditions, no breakthrough occurs during diffusion cell experiments in completely dry cores^53^. Recent work on xenon^29,48,50,51,54–57^ has similarly shown significant adsorption to zeolites in tuff. If adsorption processes are active in the field, they are expected to suppress peak tracer concentrations and delay breakthrough, particularly in boreholes where a substantial fraction of transport occurs through zeolitic tuff. The absence of adsorption in the model could therefore explain discrepancies between predicted and observed tracer arrival times and peak concentrations, particularly at boreholes where zeolitic tuff is a dominant lithology and as the early-time pressure wave diminishes.

From the above references, we expect adsorption to zeolites to be much more significant for CO_2_ and ^127^Xe than for the other gas species examined. The upper VNT units (VNT-a, VNT-1) are generally vitric tuffs^19^, and gradually increase in zeolitization with increasing depth. We would therefore expect to overpredict concentrations for CO_2_ and ^127^Xe at boreholes located within deeper units (i.e., more zeolitized) such as at GS-1, 3, and 5, assuming the permeabilities used are accurate. This is in fact observed in our simulations, however the model also overpredicts concentration of ^127^Xe at GS-2, which is hosted in VNT-1, whereas modeled CO_2_ was underpredicted. We note that, because ^127^Xe concentrations are significantly overpredicted at all proximal boreholes, and CO_2_ concentrations are both over- and under-predicted at the same boreholes, we do not expect zeolite adsorption to account for all discrepancies observed in our model. However, accounting for adsorption could improve the model’s ability to predict earlier gas breakthrough times at most boreholes. Improving field-scale gas transport models by conducting bench-scale experiments of noble gas transport through variably saturated tuffs and other rocks is an area of ongoing focus by our team.

## Conclusions

This study presents a modeling approach to understand the rapid multi-phase transport of radionuclide tracers and high-explosive (HE) byproducts in the vadose zone following an underground chemical explosion. By incorporating pre-shot predictions of permeability and pressure propagation from previous work, our flow and transport model predicted with reasonable (order-of-magnitude) accuracy the observed gas concentrations in boreholes near the explosion cavity, even considering the high degree of expected heterogeneity at the field scales examined (10s–100s of m). Our predictions using pre-shot cavity pressurization-calibrated permeability were less accurate at distal boreholes in which the pressure perturbations were negligible. These discrepancies highlight the inherent challenges in capturing site-scale heterogeneity and the limitations of model calibration based on localized datasets. This drives home the need for integrating multiple data streams (e.g., core samples, packer tests, and cavity pressurization tests) in predictions used to inform engineering decisions related to gas behavior for two future experiments in the PE series.

Field-scale subsurface transport datasets are relatively scarce, and those capturing transport driven by high-pressure conditions are even more limited, making the predictive modeling efforts presented in this work relatively unique. While our model necessarily simplifies aspects of the coupled thermo-hydrologic processes, it enables a physically consistent interpretation of field observations*.* The insights gained from this unique dataset and modeling approach have broad implications for understanding the coupling between underground explosions, transient pressure fields, and the resulting transport of hazardous materials in the vadose zone. Overall, this work represents a step forward in our ability to predict and mitigate the environmental risks associated with rapidly migrating subsurface contaminants.

## Software availability

The FEHM software^22^ used in this research (version 3.6.2) is published on Zenodo^58^ under the BSD-3 license and without any access restrictions. Computation mesh was generated using the LaGriT software^30^ v3.3.3 which is available under the BSD-3-Clause license and without any access restrictions on GitHub (https://github.com/lanl/LaGriT). Figures were made with Matplotlib version 3.2.2 (Hunter, 2007) available under the Matplotlib license at https://matplotlib.org/. Scripts used to process and plot input/output data, as well as all input files needed to generate the mesh and run the FEHM simulations are hosted in a repository on Zenodo (PrivateReviewerRepo).

## Supplementary Information

Supplementary Information 1.

Supplementary Information 2.

Supplementary Information 3.

Supplementary Information 4.

Supplementary Information 5.

Supplementary Information 6.

Supplementary Information 7.

Supplementary Information 8.

Supplementary Information 9.

Supplementary Information 10.

## Data Availability

Data for this research are not publicly available until after Feb 9, 2026, following an embargo on the public release of the data from the sponsoring government organization. After this date, data will be made available via public repository. For reproducibility, experimental data used in the analysis will be included as individual text files in the Supporting Information for editor/reviewer purposes. Description of HE byproducts data are described in “HE Byproducts Metadata User Guide_02062023.pdf”. The HE byproducts tracer data files are uploaded separately for each borehole (e.g., “HEdata_GS1.csv”). Description of the xenon data is described in “README_PE1A_Radioxenon_Concentration.txt”. The xenon tracer data file is uploaded as “XeData.csv”. The experimental data will be uploaded either as Supporting Information or as a repository before publication, depending on sponsor approval.
