# Supplementary material for: Predicting multiphase flow and tracer transport for an underground chemical explosive test
Source: Sci Rep. 2026 Feb 17;16:9431. doi: 10.1038/s41598-026-35868-w (PMC13002974; doi:10.1038/s41598-026-35868-w)
Supplement: Supplementary file 1 — Supplementary Information 1. [file 41598_2026_35868_MOESM1_ESM.pdf]

Metadata User Guidance Document –
High Explosive (HE) Byproducts Gas Analysis

This repository contains the data from the HE Byproducts gas analysis. The data was collected using NOVA gas analyzers (model: 876SMN4). Each borehole was connected to one analyzer. The gas analyzer analyzes O_2, CO, CO_2, CH_4, NH_3, and H_2 in real time. For more detail about the instrumentation see HE Byproducts As-built document.

The LYNM PE1 HE Byproducts Calibration and Tracer Tests document contains information about how the instruments were calibrated and more information about the tracer tests. Though 3 tracer tests were performed the first was just a proof of concept and the data are not included, only the data from the latter two tracer tests are included.

Background data was collected from September 8th, 2023 through October 11th, 2023, some data is missing as the server became full and there were some power outages with the remote computers.

Shot was performed on October 18th 2023 at 15:15 UTC.

Data Files:

Data files are csv files. The file name format is ID**(GS#)_yyyymmddhhmm** (start date and time of that particular file). A new file is created approximately every 48 hours and complete files are about 21.1 KB. Each files contains just the data from one of the 8 boreholes.

Data Dictionary:

Column	Value	Description	E.g.
1	Date	"yyyy-mm-ddThh-mm-ss.sss AM/PM" format, UTC date time	2023-11-15T02-26-04.342 PM
2	O2 _%	Oxygen concentration in percent (this varies with pressure and is not an accurate representation of oxygen concentrations)	19.7989
3	CO _%	Carbon monxoide concentration in percent	5.9215
4	CO2 _%	Carbon dioxide concentration in percent	0.394
5	CH4 _%	Methane concentration in percent	2.3575
6	NH3 _%	Ammonia concentration in percent	0.0929
7	H2 _%	Hydrogen concentration in percent	3.1933
8	FLOW_LPM	Flow in the line in liters per minute (the digital flow meter on unit 3 connected to GS3 is broken but flow was around 1 LPM)	0.9994
9	VAC _I HG	Vacuum Pressure in the line in inches Hg	2.2059
10	TEMP_C	Temperature of the instrument in Celsius	5.16
11	O2 _RawCount	Oxygen concentration in raw counts	41119
12	CO _RawCount	Carbon monoxide concentration in raw counts	12597

**13**	**CO2 _RawCount**	**Carbon dioxide concentration in raw counts**	**956**
**14**	**CH4 _RawCount**	**Methane concentration in raw counts**	**17367**
**15**	**NH3 _RawCount**	**Ammonia concentration in raw counts**	**56**
**16**	**H2 _RawCount**	**Hydrogen concentration in raw counts**	**659**
**17**	**FLOW_RawCount**	**Flow in raw counts**	**1865**
**18**	**VAC _RawCount**	**Vacuum Pressure in raw counts**	**678**
**19**	**TEMP_RawCount**	**Temperature in raw counts**	**136**
**20**	**SSR1_16**	**see pg. 45 of manual**	**0 0 0 1 0 1 1 1 1 1 1 1 1 1 1 1**
**21**	**ALM1_16**	**see pg. 46 of manual**	**0 0 0 0 0 0 0 0 0 0 0 0 0 0 0 0**

Appendix A - Background Data with periods of data collection and missing data

	ID 1	
	start	end
data	2023-09-08T09-37-25.500 PM	2023-09-13T03-00-03.487 AM
missing		
data	2023-09-18T02-57-59.101 AM	2023-10-04T06-23-25.096 AM
missing		
data	2023-10-09T06-07-16.589 PM	2023-10-10T09-19-16.899 PM
missing		
data	2023-10-10T09-40-49.770 PM	2023-10-10T09-59-29.617 PM
missing		
data	2023-10-11T03-43-26.165 PM	2023-10-11T07-36-59.672 PM
	ID 2	
	start	end
data	2023-09-08T09-33-41.992 PM	2023-09-13T03-00-03.414 AM
missing		
data	2023-09-18T02-58-31.755 AM	2023-10-05T05-11-28.810 PM
missing		
data	2023-10-09T06-06-39.345 PM	2023-10-10T09-19-15.174 PM
missing		
data	2023-10-10T09-41-05.463 PM	2023-10-10T09-59-30.054 PM

missing

data 2023-10-11T03-43-21.683 PM 2023-10-11T07-36-59.593 PM

ID 3

start end

data 2023-09-08T09-25-18.824 PM 2023-09-13T03-00-03.676 AM

missing

data 2023-09-18T02-58-03.520 AM 2023-09-18T10-18-56.396 PM

missing

data 2023-09-18T10-30-55.416 PM 2023-09-27T10-11-38.887 AM

missing

data 2023-09-27T10-11-45.330 AM 2023-10-10T09-19-16.554 PM

missing

data 2023-10-10T09-40-57.318 PM 2023-10-10T09-59-30.530 PM

missing

data 2023-10-11T03-43-15.189 PM 2023-10-11T07-37-00.488 PM

ID 4

start end

data 2023-09-08T09-35-27.053 PM 2023-09-13T03-00-03.801 AM

missing

data 2023-09-18T02-58-24.781 AM 2023-09-18T10-18-59.415 PM

missing

data 2023-09-18T10-30-44.625 PM 2023-10-05T05-11-28.095 PM

missing

data 2023-10-09T06-06-55.001 PM 2023-10-10T09-19-15.174 PM

missing

data 2023-10-10T09-41-20.860 PM 2023-10-10T09-59-30.451 PM

missing

data 2023-10-11T03-43-10.641 PM 2023-10-11T07-37-00.849 PM

ID 5

	start	end
data	2023-09-08T11-07-42.590 PM	2023-09-13T05-00-02.228 AM
missing		
data	2023-09-18T02-33-22.644 AM	2023-09-18T10-00-39.190 PM
missing		
data	2023-09-18T10-30-25.784 PM	2023-09-18T10-33-43.405 PM
missing		
data	2023-09-20T11-00-40.780 PM	2023-10-05T05-11-29.964 PM
missing		
data	2023-10-09T06-05-54.368 PM	2023-10-10T09-18-40.937 PM
missing		
data	2023-10-10T09-40-48.623 PM	2023-10-10T09-59-22.564 PM
missing		
data	2023-10-11T03-42-47.718 PM	2023-10-11T07-27-53.272 PM

ID 6

	start	end
data	2023-09-08T10-37-17.216 PM	2023-09-13T05-00-01.068 AM
missing		
data	2023-09-18T02-57-28.891 AM	2023-09-18T10-01-46.614 PM
missing		
data	2023-09-18T10-30-24.966 PM	2023-09-18T10-33-43.235 PM
missing		
data	2023-09-21T02-42-59.757 AM	2023-10-05T05-11-29.399 PM
missing		
data	2023-10-09T06-05-52.877 PM	2023-10-10T09-18-40.276 PM
missing		
data	2023-10-10T09-40-46.935 PM	2023-10-10T09-59-21.852 PM
missing		
data	2023-10-11T03-42-46.414 PM	2023-10-11T07-27-53.712 PM

	ID 7	
	start	end
data	2023-09-08T10-27-07.800 PM	2023-09-13T05-00-01.460 AM
missing		
data	2023-09-18T02-57-33.951 AM	2023-09-18T10-01-39.646 PM
missing		
data	2023-09-18T10-30-28.657 PM	2023-09-18T10-33-43.909 PM
missing		
data	2023-09-20T11-06-32.689 PM	2023-10-05T05-11-29.525 PM
missing		
data	2023-10-09T06-05-56.145 PM	2023-10-10T09-18-41.282 PM
missing		
data	2023-10-10T09-40-49.690 PM	2023-10-10T09-59-22.543 PM
missing		
data	2023-10-11T03-42-42.378 PM	2023-10-11T07-27-53.194 PM
	ID 8	
	start	end
data	2023-09-08T10-35-09.902 PM	2023-09-13T05-00-02.746 AM
missing		
data	2023-09-18T02-57-38.868 AM	2023-09-18T10-01-47.626 PM
missing		
data	2023-09-18T10-30-32.999 PM	2023-09-18T10-33-44.859 PM
missing		
data	2023-09-20T10-58-50.759 PM	2023-10-05T03-09-09.497 AM
missing		
data	2023-10-05T11-06-32.651 AM	2023-10-05T05-11-29.572 PM
missing		
data	2023-10-09T06-05-50.882 PM	2023-10-10T09-18-41.282 PM
missing		

data	2023-10-10T09-40-51.677 PM	2023-10-10T09-59-22.198 PM
missing		
data	2023-10-11T03-42-41.736 PM	2023-10-11T07-27-53.304 PM
