# Supplementary material for: Predicting multiphase flow and tracer transport for an underground chemical explosive test
Source: Sci Rep. 2026 Feb 17;16:9431. doi: 10.1038/s41598-026-35868-w (PMC13002974; doi:10.1038/s41598-026-35868-w)
Supplement: Supplementary file 9 — Supplementary Information 9. [file 41598_2026_35868_MOESM9_ESM.pdf]

Scientific Reports

Supporting Information for

Predicting multiphase flow and tracer transport for an underground chemical explosive test

J. P. Ortiz[1], D. D. Lucero[1], E. Rougier[1], E. E. Knight[1], S. M. Bourret[1], B. G. Fritz[2], M. A., Bodmer[3], J. E. Heath[3], C. W. Neil[1], H. Boukhalfa[1], K. L. Kuhlman[3], S. Otto[1], S. Ezzedine[4], B. L. Roberts[3], R. C. Choens[3], G. A. Zyvoloski[5], P. H. Stauffer[1], and PE1 Experimental Team[*]

[1]Los Alamos National Laboratory, Los Alamos, NM, USA.

[2]Pacific Northwest National Laboratory, Richland, WA, USA.

[3]Sandia National Laboratories, Albuquerque, NM, USA.

[4]Lawrence Livermore National Laboratory, Livermore, CA, USA.

[5]Neptune and Company, Los Alamos, NM, USA.

*A list of authors and their affiliations appears at the end of the paper.

Contents of this file

Introduction
Figures S1 to S6

Introduction

This Supporting Information document contains additional information about the study.

Figure S1 presents the full geologic framework model used in the simulations. Figure S2 presents the full set of concentration time series results for all borehole gas sensors based on the simulation using permeabilities calibrated from the pre-shot cavity pressurization test. Figure S3 presents the full set of concentration time series results for all borehole gas sensors based on the simulation using permeabilities calibrated from the post-shot pressure data collected during the PE1-A chemical explosive test. Figure S4 presents the bias calculations for all borehole gas sensors based on the simulation using permeabilities calibrated from the post-shot pressure data collected during the PE1-A chemical explosive test. Figure S5 presents several time slices of the water saturation surrounding the cavity in response to the pressure wave. Figure S6 is a schematic showing how properties were modified in the pore crush shell surrounding the cavity.

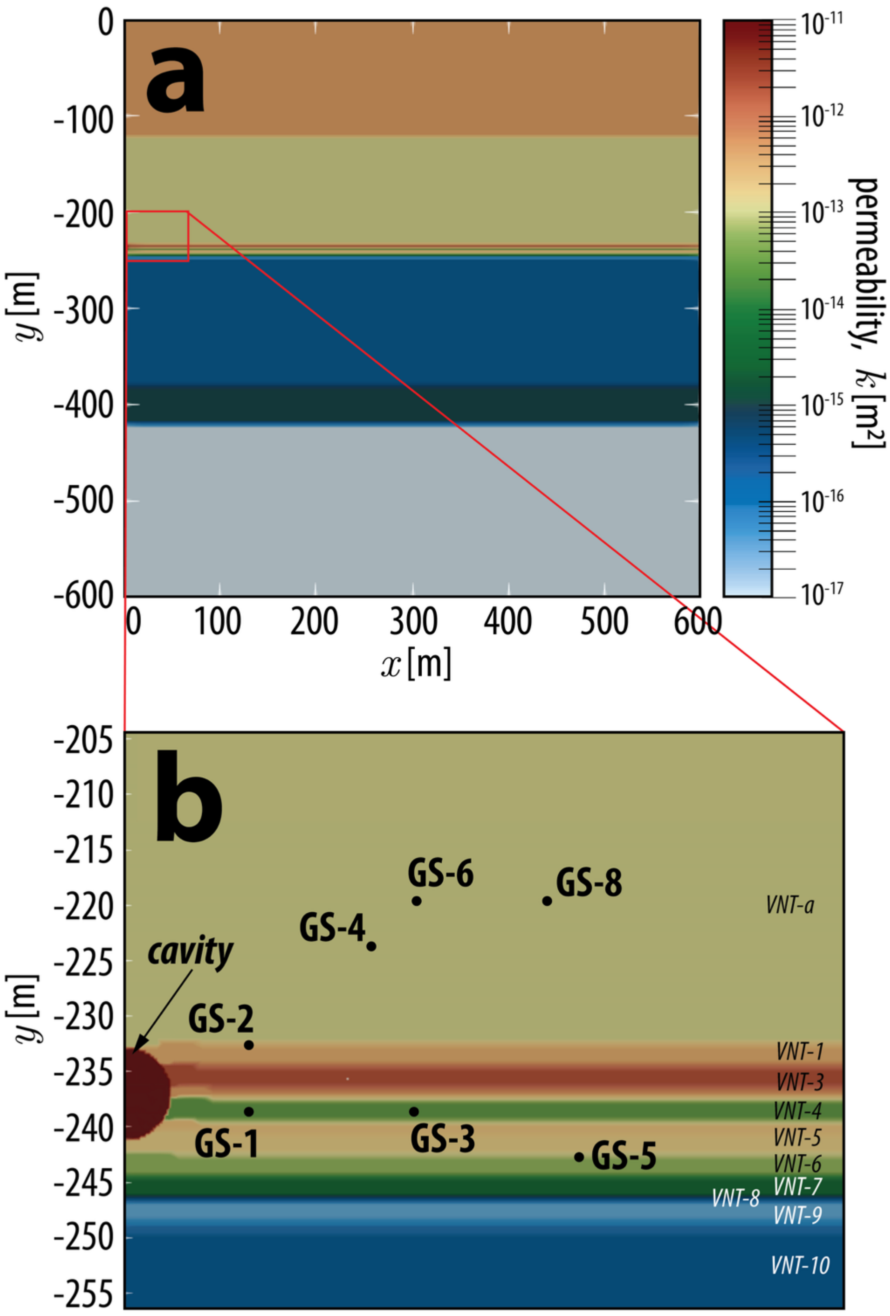

Figure S1. Hydrogeologic model domain with calibrated permeabilities from pre-shot measurements presented in Lucero et al. (2025). (a) Full model domain with red box showing extent of zoomed-in portion of domain surrounding the cavity, and (b) zoomed-in portion of domain surrounding the cavity working point. Permeability values are listed in Table S2. Four VNT units contain gas sampling (GS) boreholes: VNT-1 (GS-2), VNT-5 (GS-1 and 3), VNT-8 (GS-5) and VNT-a (GS-4, 6 and 8).

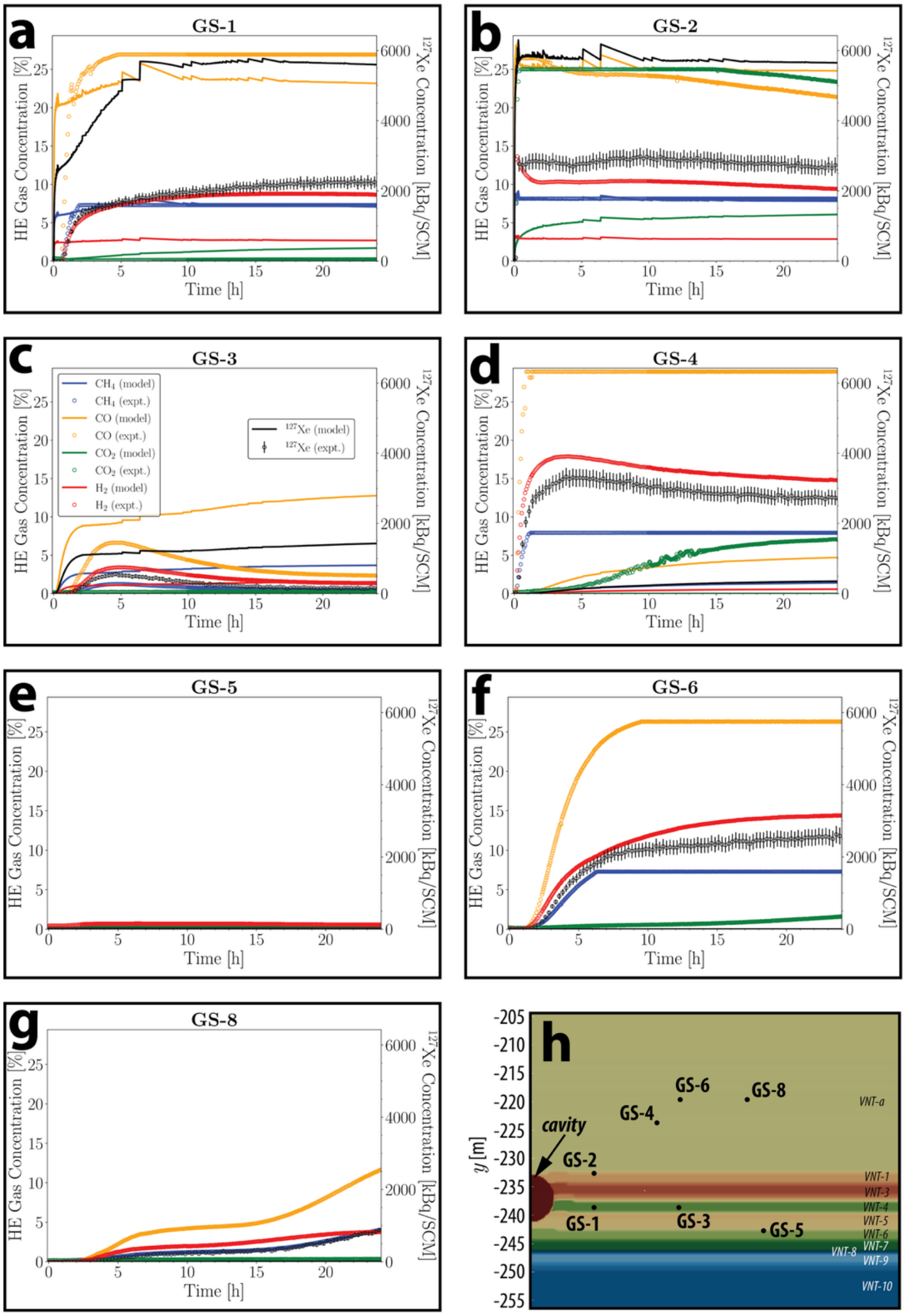

Figure S2. Composite plot of simulated gas concentrations using permeability values from pre-shot calibration and experimental gas concentrations at each borehole: (a) GS-1, (b) GS-2, (c) GS-3, (d) GS-4, (e) GS-5, (f) GS-6, (g) GS-8, and (h) zoomed in section of model domain showing gas sampling borehole locations. Note that ^{127}Xe concentrations (black) are plotted against the right y-axis in units of kBq/SCM.

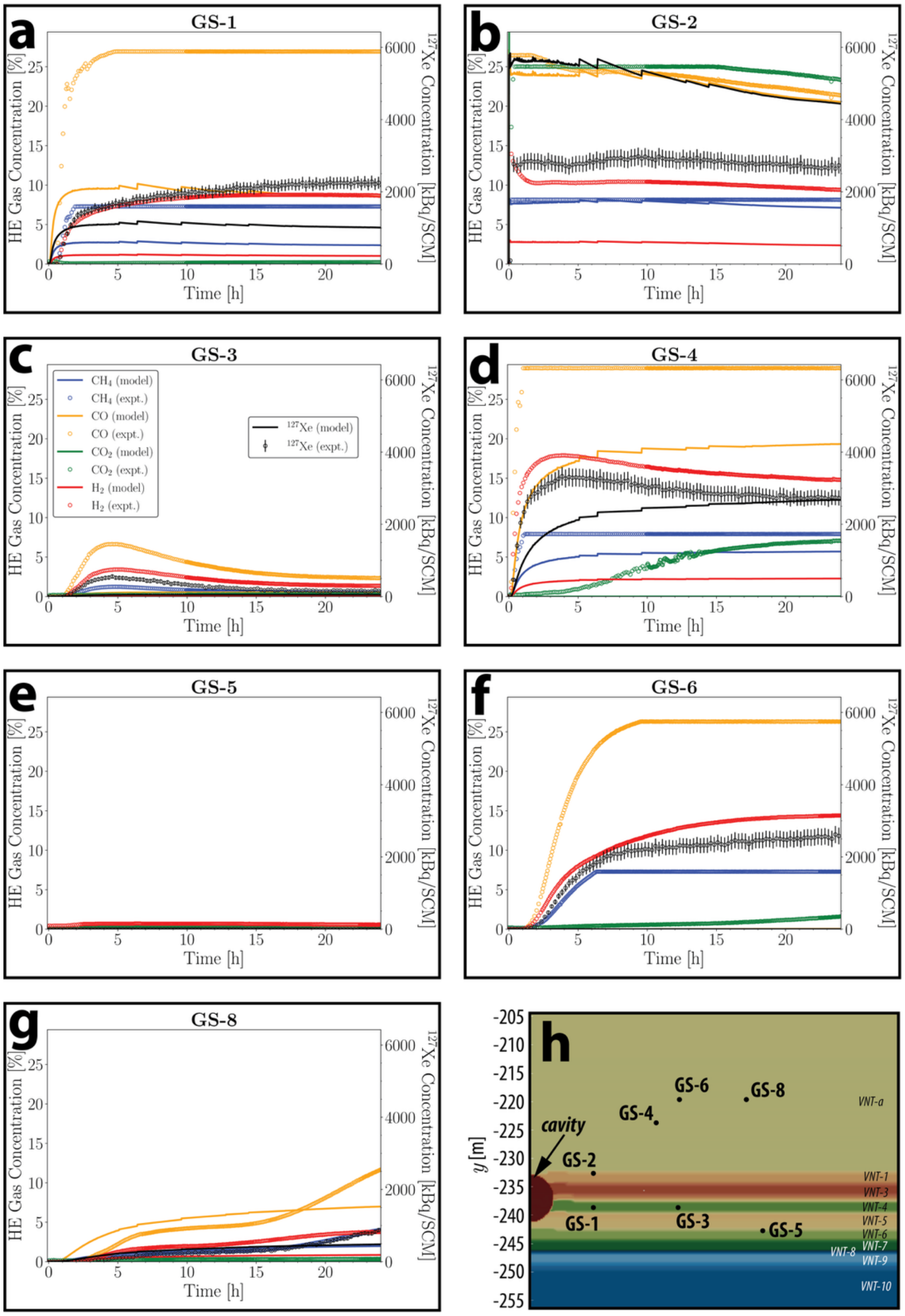

Figure S3. Composite plot of simulated gas concentrations using permeability values from post-shot calibration and experimental gas concentrations at each borehole: (a) GS-1, (b) GS-2, (c) GS-3, (d) GS-4, (e) GS-5, (f) GS-6, (g) GS-8, and (h) zoomed in section of model domain showing gas sampling borehole locations.

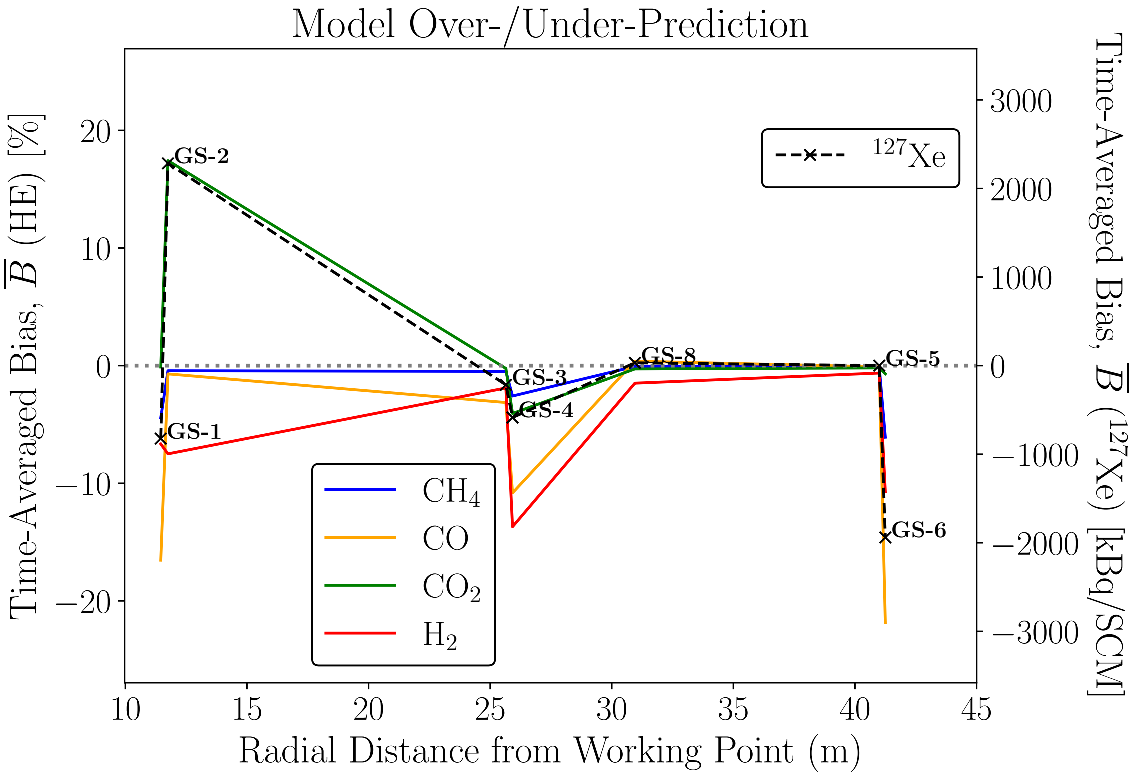

Figure S4. Representation of model over-/under-prediction of tracer concentration as a function of lateral distance from the working point as calculated using permeabilities derived from a post-shot permeability calibration. HE byproducts are represented by solid colored lines corresponding to the left ***y***-axis [%], whereas ^{127}Xe is represented by the dashed line corresponding to the right ***y***-axis [kBq/SCM].

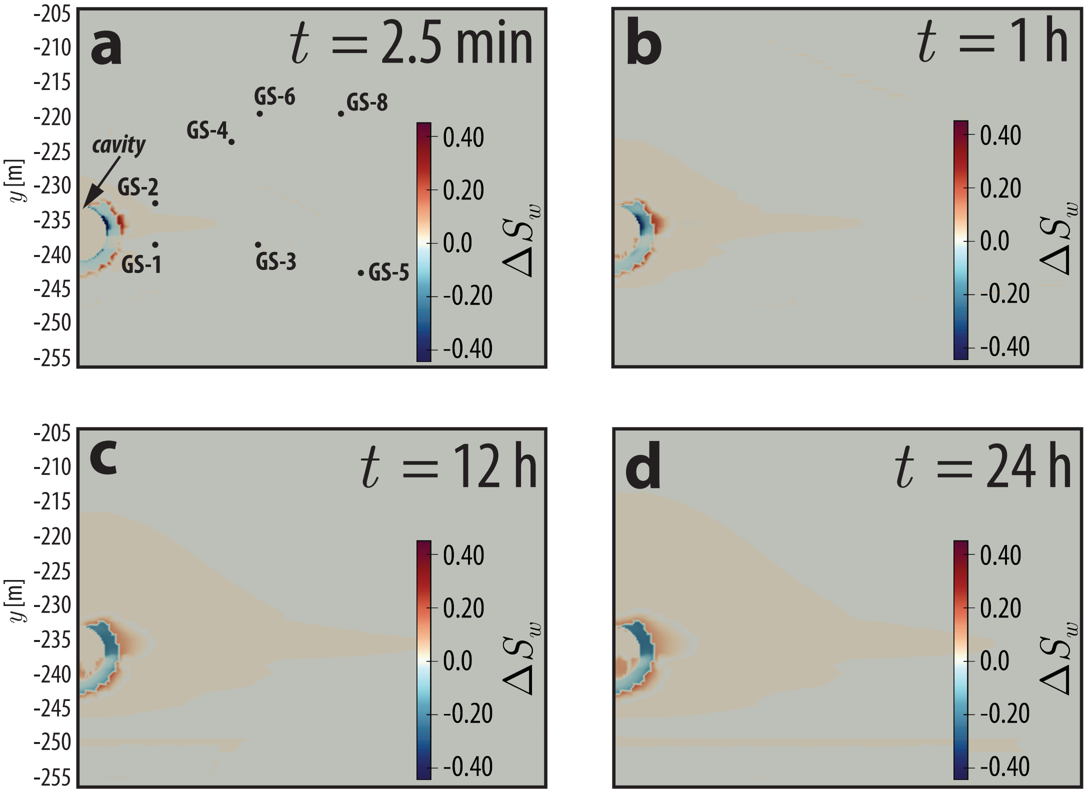

Figure S5. Multiple time slices of water saturation surrounding the working point, which moves according to our relative permeability function in response to excess pressure buildup within the cavity. Colors indicate the difference in saturation at the given time relative to initial conditions: $\Delta S_w = S_w(\mathrm{t}) - \mathrm{S_w}(t = 0)$.

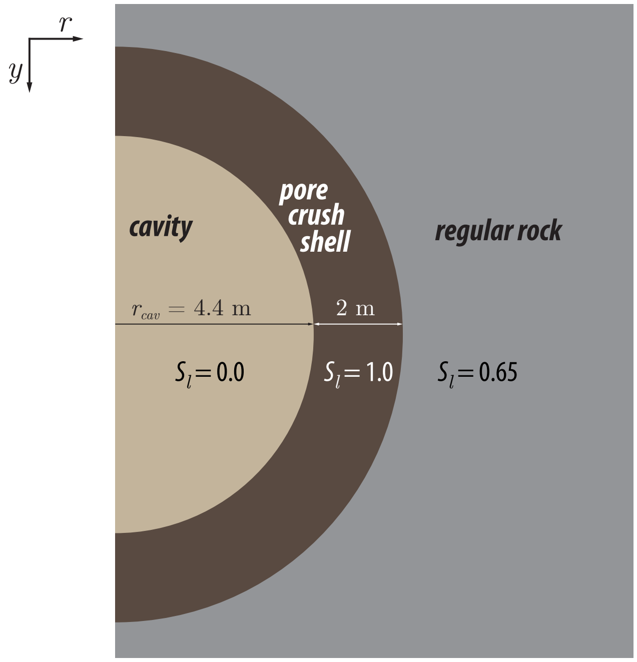

Figure S6. Schematic describing the pore crush shell. Pore crush derived from hydrodynamics simulations makes air-filled porosity become negligible, such that rock in the approximately 2 m ring surrounding the cavity becomes fully saturated (liquid saturation S_l =1.0).
